# Low Visceral Adipose Tissue Predicts the Outcome of Neoadjuvant Chemotherapy for Colorectal Liver Metastases: A Multicentre Real‐World Study

**DOI:** 10.1002/jcsm.13785

**Published:** 2025-04-01

**Authors:** Yizhen Chen, Hangdong Jia, Rong Ye, Zhenyuan Zhou, Weijie Chen, Ming Zheng, Yuanyuan Zheng

**Affiliations:** ^1^ Department of Geriatric Medicine, Fujian Key Laboratory of Geriatrics Diseases, Fujian Provincial Center for Geriatrics, Fujian Provincial Hospital, Shengli Clinical Medical College of Fujian Medical University Fuzhou University Affiliated Provincial Hospital Fuzhou Fujian China; ^2^ General Surgery, Cancer Center, Department of Hepatobiliary & Pancreatic Surgery and Minimally Invasive Surgery, Zhejiang Provincial People's Hospital, Affiliated People's Hospital Hangzhou Medical College Hangzhou Zhejiang China; ^3^ Department of Colorectal Surgery The First Affiliated Hospital of Fujian Medical University Fuzhou Fujian China; ^4^ Anorectal Surgical Department Hangzhou Red Cross Hospital Hangzhou Zhejiang China; ^5^ Department of Hepatobiliary Pancreatic Surgery The First Hospital of PuTian City Putian Fujian China

**Keywords:** colorectal cancer, liver metastasis, neoadjuvant chemotherapy, visceral adipose tissue, visceral obesity

## Abstract

**Background:**

Visceral obesity (VO), associated with excessive visceral adipose tissue (VAT), has been extensively studied in cancer. However, whether low VAT can predict the prognosis of colorectal liver metastases (CRLM) undergoing neoadjuvant chemotherapy (NAC) remains unknown.

**Methods:**

This multicentre real‐world cohort study analysed data from initially resectable CRLM patients who received NAC. The predictive effect of VAT on progression‐free survival (PFS) and overall survival (OS) was evaluated using restricted cubic splines (RCS). VAT was categorized into low/normal VAT and VO groups using X‐tile. The prognostic differences were further assessed through Kaplan–Meier (KM) analysis. The impact of changes in VAT (ΔVAT) after NAC was evaluated.

**Results:**

Among 1524 CRLM patients, 1105 patients (72.51%) were under 65 years old, with a median VAT of 84.00 (36.24–148.00) cm^2^. Of all patients, 804 (52.76%) were female. A U‐shaped nonlinear relationship was observed between VAT and both PFS/OS (*p* < 0.001). Compared with the normal VAT, both low VAT and VO groups showed worsened PFS and OS (*p* < 0.05). The 3‐year PFS rate was 31.6%, 69.0% and 42.0% in the low, normal VAT and VO groups (*p* < 0.05). The 3‐year OS rate was 76.4%, 88.9% and 79.4% in the low, normal VAT and VO groups (*p* < 0.05). There was also a nonlinear relationship between VAT and NAC‐related adverse events, objective response rate and postoperative complications (*p* < 0.001). An increase in ΔVAT in the low VAT group was associated with better PFS and OS (*p* < 0.05). In the VO group, both increases and decreases in ΔVAT were associated with worsened PFS and OS (*p* < 0.05).

**Conclusions:**

This study is the first to reveal that low VAT and VO can predict PFS and OS in CRLM patients undergoing NAC. Baseline VAT and ΔVAT may serve as important indicators for risk stratification and personalized treatment in CRLM patients.

## 1 Introduction

The incidence of colorectal cancer (CRC) is increasing annually, and mortality rate remains high [1]. Colorectal liver metastasis (CRLM) occurs in more than half of CRC, significantly worsening long‐term prognosis [2]. Hepatectomy is the only potentially curative treatment for CRLM, yet the high postoperative recurrence rate poses a clinical challenge in improving the prognosis of initially resectable CRLM patients.

Neoadjuvant chemotherapy (NAC) is a crucial component of treatment strategies for CRLM. The use of chemotherapy before surgery aims to reduce tumour size, increase resectability and assess the sensitivity to chemotherapy [3, 4]. Currently, NAC improves progression‐free survival (PFS) in CRLM but has no effect on overall survival (OS) [5]. Additionally, significant individual variability exists among CRLM patients, with some experiencing disease progression or chemotherapy‐related liver injury during NAC, thereby losing the chance of cure. Developing new predictive methods to efficiently and conveniently identify specific CRLM populations that may benefit from NAC is essential for personalized treatment.

The predictive efficiency of body composition on cancer has gained increasing recognition [6, 7]. Traditionally, BMI has been used to assess obesity. However, a certain proportion of high‐BMI patients exhibit metabolic health with low levels of adipose tissue, a phenomenon known as the obesity paradox. As research into human body composition deepens, it is now widely accepted that BMI does not accurately represent body fat content [8]. As the key component of body composition, visceral obesity (VO) caused by excessive visceral adipose tissue (VAT) has been extensively studied in promoting cancer [9, 10]. VAT has emerged as a crucial factor in the complex landscape of cancer research. VAT may be a novel and non‐invasive way to identify aggressive renal tumour patients [11]. Increased VAT is linked to the higher risk of developing various cancers [12]. Understanding the multifaceted role of VAT in cancer is essential for unravelling the complex mechanisms underlying cancer development and for identifying potential therapeutic targets. However, previous studies have simply classified VAT into VO and non‐VO categories, overlooking the impact of low VAT on cancer prognosis [13, 14]. Some research has shown that a low BMI is linked to higher rates of tumour progression and an elevated risk of mortality in CRC [15]. It is important to note that VAT is the tissue with both immune and nutritional functions. Currently, research on the impact of low VAT on cancer is limited, highlighting the need for precise subdivision of VAT values to better understand the predictive role in NAC‐treated CRLM.

It remains unclear whether baseline VAT affects the response to NAC, postoperative complications and long‐term prognosis in CRLM patients and whether changes in VAT (ΔVAT) after NAC influence prognosis. Abdominal imaging, including computed tomography (CT), is routinely used to evaluate CRLM during NAC. This study aims to explore whether VAT can predict the outcomes of CRLM patients undergoing NAC. The goal is to provide more valuable predictive indicators for personalized treatment.

## 2 Materials and Methods

### 2.1 Grouping and Study Population

This study collected and analysed baseline, chemotherapy, surgical and prognosis data from the multicentre real‐world cohort of CRLM patients treated with NAC. Patients with initially resectable CRLM were analysed from five tertiary medical centres: Fujian Provincial Hospital, the First Affiliated Hospital of Fujian Medical University, Zhejiang Provincial People's Hospital, Hangzhou Red Cross Hospital and Putian First Hospital, between January 2010 and January 2020. The patients enrolled in this study had undergone a minimum of two cycles of NAC. Initial resectability was assessed by a multidisciplinary team (MDT). The ethics committees of the five tertiary medical centres approved this multicentre study.

Inclusion criteria were as follows: (1) age ≥ 18 years; (2) histologically confirmed CRLM; (3) MDT determination that the primary tumour was resected or resectable, and liver metastases were resectable; (4) abdominal CT scans before NAC and surgery; (5) received NAC combined with curative surgery; and (6) intact major organ function. Exclusion criteria were as follows: (1) fewer than two cycles of NAC; (2) history of malignancy other than CRC; (3) presence of extrahepatic metastases; (4) emergency surgery; (5) missing key data such as CT scans; and (6) loss to follow‐up.

### 2.2 Comprehensive Treatment Procedure and Follow‐Up

Following relevant guidelines, the MDT provided standardized treatment plans for all initially resectable CRLM patients. The MDT and patients jointly considered various factors (e.g., adverse prognostic factors and patient physical condition) in deciding on chemotherapy regimens. The five included tertiary medical centres employed classic NAC regimens (CapeOX, mFOLFOX6, FOLFIRI and FOLFOXIRI). Considering potential chemotherapy‐related liver damage, NAC were limited to six cycles or 3 months [16]. The response to chemotherapy was evaluated via CT during NAC. After NAC, a repeat CT scan was used to reassess liver metastases. Based on comprehensive evaluation by the MDT, patients underwent curative surgery by experienced surgical teams.

Follow‐up for CRLM patients was conducted through telephone, outpatient visits and hospital record systems. In brief, all CRLM patients underwent postoperative carcinoembryonic antigen (CEA) and imaging (including chest CT, abdominal ultrasound and abdominal‐pelvic CT). Annual endoscopy was recommended postoperatively. The follow‐up procedures at the five tertiary medical centres adhered to international guidelines and consensus.

### 2.3 Measurement of VAT

This study collected baseline (pre‐NAC) and post‐NAC CT images, using classic CT scanning techniques to measure VAT. Specifically, two radiologists and hepatobiliary surgeons, blinded to patient information, delineated VAT at the L3 on CT images, an area representative of overall body composition [17]. Using VAT at L3 for analysis has been a common and standard method in many classical studies [18, 19]. The average value from the two analysts was used in subsequent analyses. If there was a significant discrepancy between the two researchers, a third researcher was involved to reassess. VAT was defined within the range of −150 to −50 Hounsfield Unit (HU). These analyses were supported by SliceOmatic, version 5.0 (Tomo Vision) [20], a validated method for precise assessment of VAT.

### 2.4 Study Endpoints and Related Definitions

The primary endpoints were the association between baseline VAT and 3‐year OS and PFS in CRLM patients. Secondary endpoints included the relationship between baseline VAT and objective response rate (ORR), chemotherapy‐related adverse events (AEs), postoperative complications and the impact of post‐NAC changes in VAT (ΔVAT) on prognosis.

NAC was defined as receiving chemotherapy according to the MDT before local cure treatment (CapeOX, mFOLFOX6, FOLFIRI and FOLFOCIRI were included in this study) [21]. ΔVAT % was defined as the percentage change in VAT before and after NAC. Initial resectability was determined by the MDT based on patient condition, the possibility of curative resection of all liver metastases and the preservation of sufficient functional liver tissue post‐surgery. PFS was defined as the time from the start of NAC to disease recurrence or death in initially resectable CRLM patients. OS was defined as the time from the start of NAC to death or the censored. NAC‐related AEs were assessed and managed according to the Common Terminology Criteria for Adverse Events (CTCAE) version 5.0 [22]. Only grade 3 or higher AEs were recorded because of the nature of retrospective study. Postoperative complications were graded using the Clavien–Dindo (CD) classification system. The response of CRLM to NAC was evaluated according to RECIST 1.1 criteria. R0 resection was defined as complete tumour resection with negative pathological margins. The staging, chemotherapy response and VAT of CRLM were assessed by radiologists and hepatobiliary surgeons. Based on the literature [13, 23, 24], the results from RCS and X‐Tile (see the results section for details), we defined VAT > 100 cm^2^ as the VO group. VAT ≤ 19 cm^2^ was defined as the low VAT group (*N* = 251), VAT between 19 and 100 cm^2^ as the normal VAT group (*N* = 659) and VAT > 100 cm^2^ as the VO group (*N* = 614).

### 2.5 Statistical Analysis

In statistical models, treating VAT as a binary variable could force the risk of outcomes to be uniform within a VAT category, leading to discontinuities in prognostic risk between different categories. We introduced the new concept of low VAT and analysed it as a continuous variable. Other prognostic factors were stratified in Cox proportional hazards and logistic regression models. These factors included baseline age, gender, Eastern Cooperative Oncology Group Performance Status (ECOG‐PS), T stage and lymph node stage of the primary tumour, baseline CEA levels, maximum liver metastasis diameter, pathological grade, synchronous metastases, NAC regimen, NAC cycles and the number of liver metastases. The study used restricted cubic splines (RCS) to visually assess the relationship between hazard ratios (HRs), odds ratios (ORs) and VAT (with five knots) in diagnostic (logistic regression) and prognostic (Cox proportional hazards) models [15]. In summary, we evaluated the impact of VAT on PFS, OS and other secondary outcomes by integrating various prognostic factors in univariate and multivariate models (Cox proportional hazards model and logistic regression model). We applied the proportional hazards assumption (PH) and variance inflation factor (VIF) to assess the model's validity and check for multicollinearity. RCS were used to visually assess the relationship between VAT and various outcomes, considering both clinical significance and potential nonlinearity.

Continuous variables were presented as mean ± standard deviation if normally distributed, or if not as median (interquartile range). After determining the impact of VAT on prognosis via RCS analysis, VAT was categorized into low VAT, normal VAT and VO groups. The analysis methods are detailed in the results section, combining RCS and X‐tile. The optimal cut‐off values of VAT were determined using X‐tile (Version 3.6.1, USA) [25]. Kaplan–Meier (KM) analysis was used to visually assess the impact of VAT on PFS and OS in CRLM patients across the three groups. A *p*‐value of less than 0.05 was considered statistically significant. R (version 4.2.1), survival [3.3.1], rms [6.4.0], ggplot2 [3.3.6] and plotRCS [0.1.3] were used for RCS analysis. KM survival analysis and Wilcoxon rank sum tests were performed using SPSS software (version 26, SPSS Inc., Chicago, IL, USA). The first author was responsible for the statistics.

## 3 Results

### 3.1 Clinicopathological Characteristics

**FIGURE 1:**
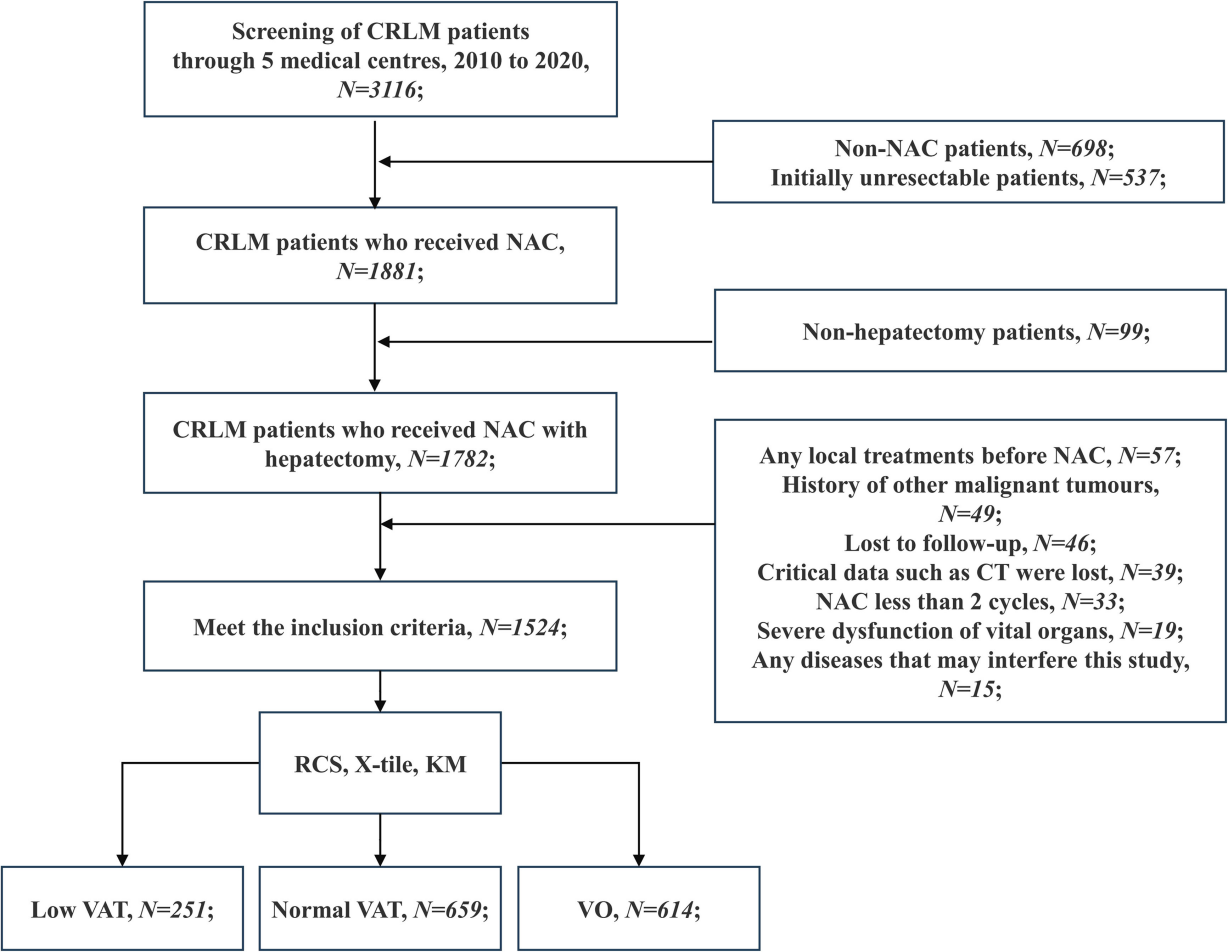
Flow chart of this research. CRLM: colorectal liver metastasis; NAC: neoadjuvant chemotherapy; RCS: restricted cubic spline; KM: Kaplan–Meier.

Our multicentre real‐world cohort study retrospectively collected and analysed data from 3116 CRLM patients across five medical centres between January 2010 and January 2020. After excluding patients who did not receive NAC, those with initially unresectable CRLM, those who did not undergo hepatectomy after NAC and so on, a total of 1524 initially resectable CRLM patients were included in the final cohort (Figure 1). All patients received NAC combined with hepatectomy, with primary lesion resection combined with hepatectomy being allowed. Additionally, complete CT scan data before NAC and before surgery were available for all patients. In the overall cohort, 1105 patients (72.51%) were under 65 years old, with a median BMI of 24.29 (20.71–28.05) kg/m^2^, and the median VAT was 84.00 (36.24–148.00) cm^2^ (Table 1).

**TABLE 1:** Baseline characteristics of the including CRLM patients.

Variables	All patients	
	No.	%
*N*	1524	
Age (years)		
< 65	1105	72.51
≥ 65	419	27.49
Baseline BMI (kg/m^2^)	24.29 (20.71–28.05)	
Baseline VAT (cm^2^)	84.00 (36.24–148.00)	
Gender		
Female	804	52.76
Male	720	47.24
ECOG		
0–1	1157	75.92
2–3	367	24.08
Location of primary cancer		
Rectum	693	45.47
Colon	831	54.53
T stage of primary tumour		
T1–T2	558	36.61
T3–T4	966	63.39
LN metastasis of primary tumour		
N0	638	41.86
N+	886	58.14
Timing of metastasis		
Metachronous	870	57.09
Synchronous	654	42.91
CEA at diagnosis, ng/mL		
≤ 200	1321	86.68
> 200	203	13.32
Number of liver metastases		
= 1	722	47.38
*≥* 2	802	52.62
Largest diameter (cm)		
< 5	1028	67.45
≥ 5	496	32.55

Abbreviations: CEA: carcinoembryonic antigen; ECOG PS: Eastern Cooperative Oncology Group Performance Status; LN: lymph node;

### 3.2 Chemotherapy and Pathological Information

All patients with initially resectable CRLM completed at least two cycles of NAC. In the overall cohort, 48.49% of patients received Cape OX, 31.96% received mFOLFOX6, 9.97% received FOLFIRI and 9.58% received FOLFOXIRI (Table 2). The median cycles of NAC were 5.00 (4.00–6.00). The ORR of CRLM to NAC was 52.62%. The rate of good and moderate histological grading was 52.56%. The non‐R0 resection rate was 6.30%. A total of 779 patients (51.11%) did not receive adjuvant chemotherapy postoperatively. The median cycles of adjuvant chemotherapy were 5.00 (3.00–7.00).

**TABLE 2:** Chemotherapy and pathological outcomes of CRLM patients.

Variables	All patients	
	No.	%
NAC regimen		
CapeOX	739	48.49
mFOLFOX6	487	31.96
FOLFIRI	152	9.97
FOLFOXIRI	146	9.58
NAC cycles	5.00 (4.00–6.00)	
Response to NAC		
CR + PR	802	52.62
SD + PD	722	47.38
Histological grade		
Well‐moderate	801	52.56
Poorly undifferentiated	723	47.44
Resection status		
R0	1428	93.70
R1 + R2	96	6.30
Postoperative chemotherapy		
Yes	745	48.88
No	779	51.11
Postoperative chemotherapy cycles	5.00 (3.00–7.00)	

Abbreviations: CR: complete response; PD: progressive disease; PR: partial response; SD: stable disease.

### 3.3 Perioperative Information

All patients underwent radical hepatectomy (Table 3). A total of 402 patients (26.38%) with initially resectable CRLM underwent synchronous radical resection of the primary tumour combined with radical hepatectomy. The mean operative time for all patients was 174.53 ± 50.08 min, the median intraoperative blood loss was 360.00 (220.25–499.00) mL, and the length of postoperative hospital stay (LOS) was 8.45 ± 2.86 days. The overall postoperative complication in CRLM patients was 23.10%, with CD grade III–V complications occurring in 86 patients (5.64%).

**TABLE 3:** Perioperative outcomes of CRLM patients.

Variables	All patients	
	No.	%
Simultaneous resection of CRC and liver metastasis; *N* (%)	402	26.38
Operative time (min)	174.53 ± 50.08	
Intraoperative blood loss (mL)	360.00 (220.25–499.00)	
LOS (days)	8.45 ± 2.86	
Postoperative complications; *N* (%)^a^	352	23.10
Major complications (Clavien III–V); *N* (%)	86	5.64

Abbreviation: LOS: length of hospital stay.

^a^ According to the Clavien–Dindo classification.

### 3.4 Analysis of VAT and △VAT on Prognosis

**FIGURE 2:**
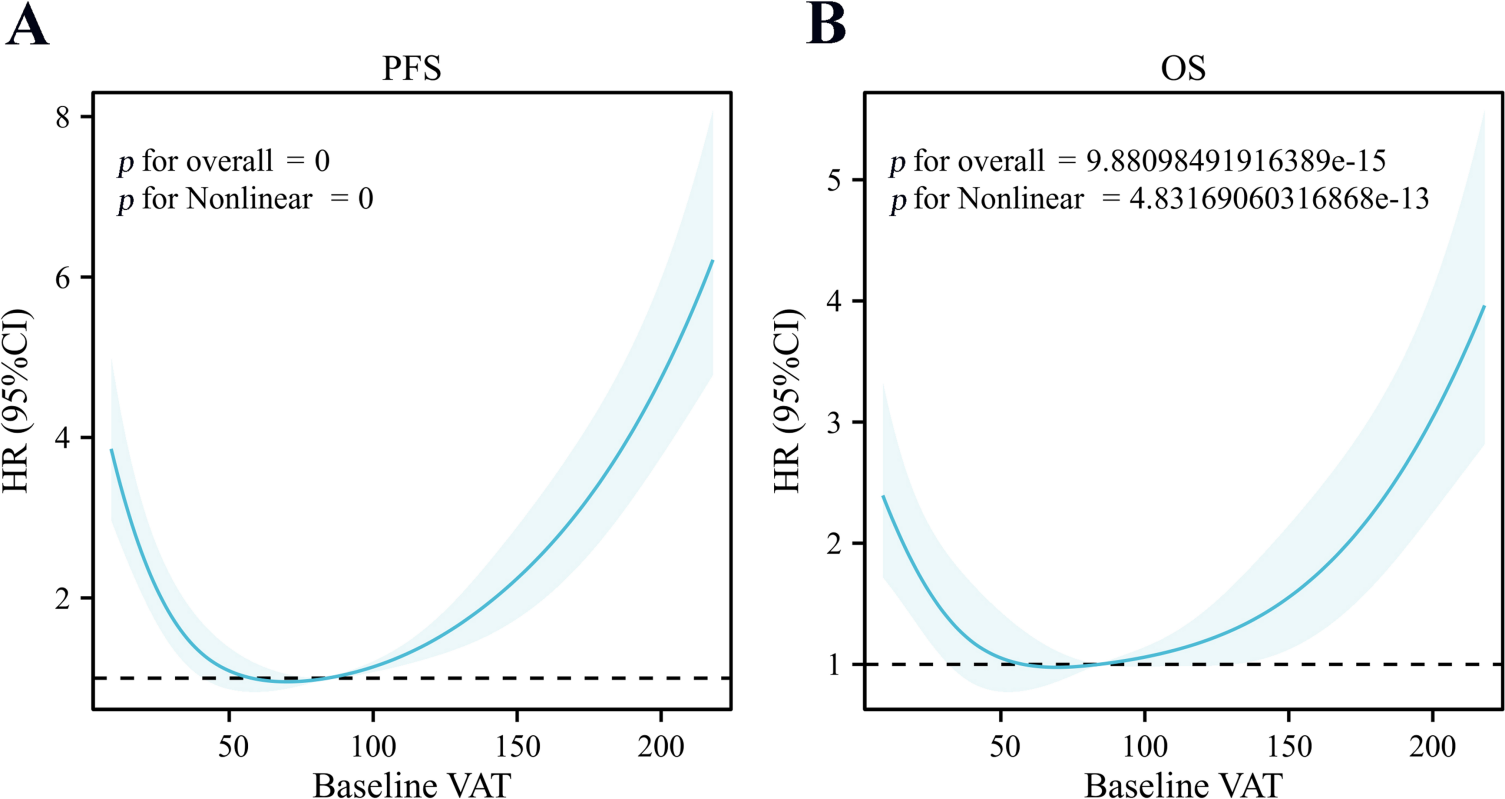
RCS (multivariate model) was used to analyse the relationship between VAT and OS/PFS. A showed a nonlinear relationship between PFS and VAT; B showed a nonlinear relationship between OS and VAT. RCS: restricted cubic spline; VAT: visceral adipose tissue.

As of December 2023, the median follow‐up time for the 1524 initially resectable CRLM patients was 38.0 (28.00–53.00) months. A total of 840 CRLM patients (55.12%) experienced recurrence or metastasis, and 490 patients (32.15%) died. We first used RCS curves to show the correlation between baseline VAT and both PFS/OS in CRLM patients undergoing NAC combined with hepatectomy. We found that the RCS curves of baseline VAT with PFS and OS both followed a U‐shaped risk trajectory (Figure 2). There was a nonlinear relationship between VAT and both PFS/OS (*p* nonlinear < 0.001), and the overall model was significant (*p* overall < 0.001).

**FIGURE 3:**
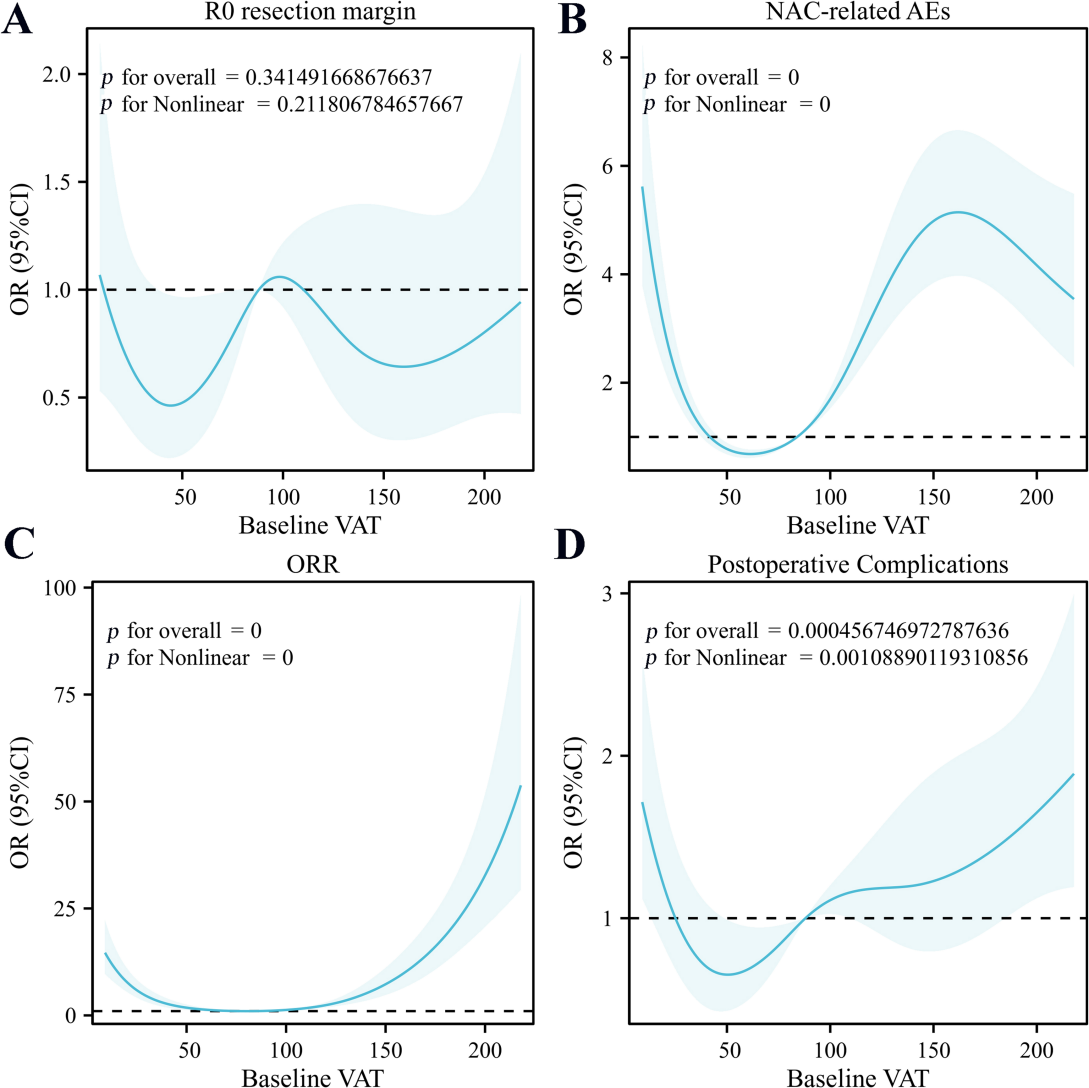
RCS (multivariate model) was used to analyse the relationship between VAT and secondary endpoint. A showed no relationship between R0 resection margin and VAT; B showed a nonlinear relationship between NAC‐related AEs and VAT; C showed a nonlinear relationship between ORR and VAT; D showed a nonlinear relationship between postoperative complications and VAT. RCS: restricted cubic spline; VAT: visceral adipose tissue; AEs: adverse events; ORR: objective response rate.

After establishing the correlation between baseline VAT and long‐term prognosis, we further explored the relationship between baseline VAT and short‐term prognosis. We found no correlation between baseline VAT and R0 resection (*p* overall > 0.05) (Figure 3A). However, there was a nonlinear relationship between baseline VAT and NAC‐related AEs, ORR and postoperative complications (*p* nonlinear < 0.001), and the overall model was significant (*p* overall < 0.001) (Figure 3B–D).

Because of the considerable variability in baseline VAT among different patients, it would be scientifically inappropriate to analyse the prognostic impact of post‐NAC changes in VAT (△VAT) without stratification. Moreover, after confirming the nonlinear relationship between VAT and PFS/OS, simply dividing VAT into two groups would not be statistically sound. Therefore, we divided VAT into three groups: VO group, normal VAT group and low VAT group. There was no consensus on the specific cut‐off point for VO in previous literature (100 cm^2^ or 130 cm^2^) [13, 23, 24]. Based on the results from the literature and RCS (Figures 2 and 3), we defined VAT > 100 cm^2^ as the VO group. We used X‐Tile to calculate the optimal cut‐off value for VAT ≤ 100 cm^2^, finding that VAT = 19 cm^2^ significantly distinguished low VAT from normal VAT (Figure S1). Therefore, VAT ≤ 19 cm^2^ was defined as the low VAT group (*N* = 251), VAT between 19 and 100 cm^2^ as the normal VAT group (*N* = 659) and VAT > 100 cm^2^ as the VO group (*N* = 614).

**FIGURE 4:**
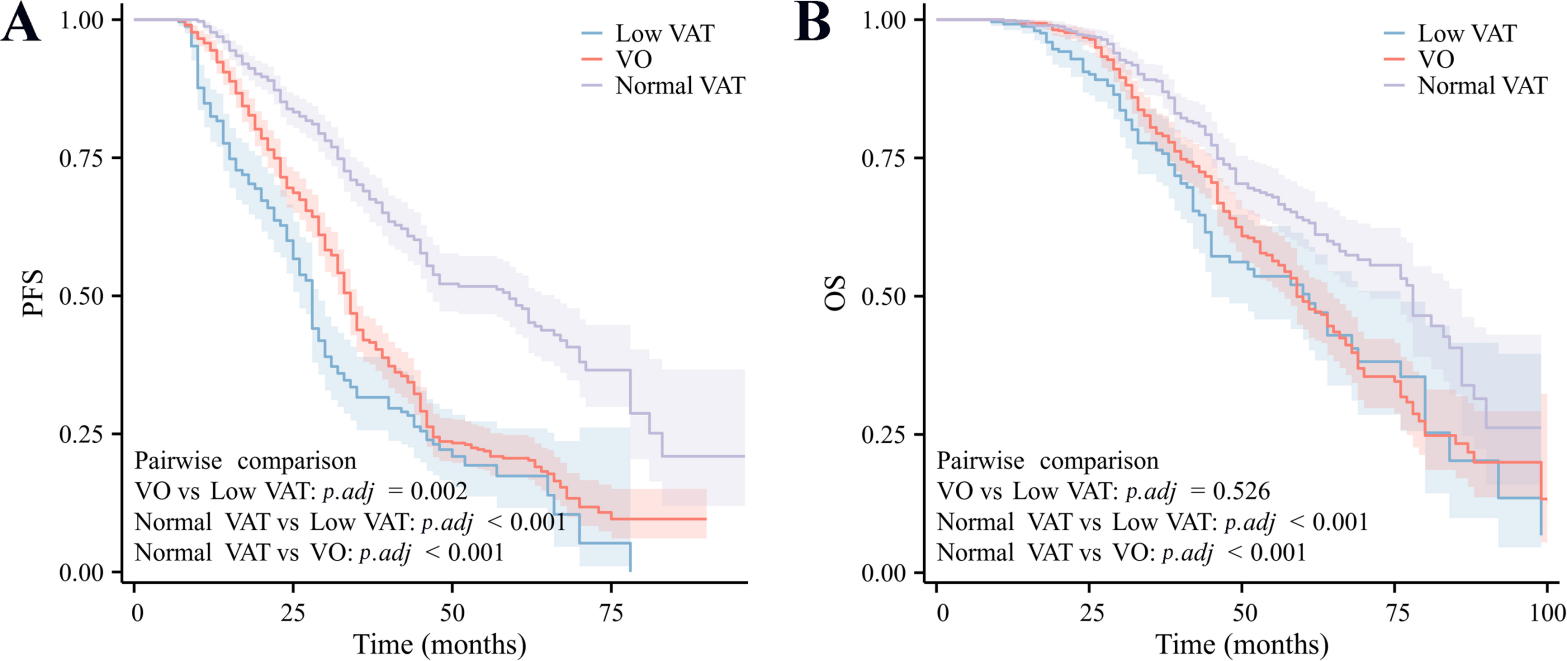
KM survival curve of PFS and OS. A was PFS, and B was OS.

We performed KM analysis on the three groups. The median PFS for the overall cohort was 38.00 months, with 28.00 months in the low VAT group, 59.00 months in the normal VAT group and 34.00 months in the VO group (Figure 4A) (Log‐rank; *p* < 0.001). The 3‐year PFS rate was 31.6% in the low VAT, 69.0% in the normal VAT and 42.0% in the VO. The median OS for the overall cohort was 65.00 months, with 61.00 months in the low VAT group, 78.00 months in the normal VAT group and 59.00 months in the VO group (Figure 4B) (Log‐rank; *p* < 0.001). The 3‐year OS rate was 76.4% in the low VAT group, 88.9% in the normal VAT group and 79.4% in the VO group.

**FIGURE 5:**
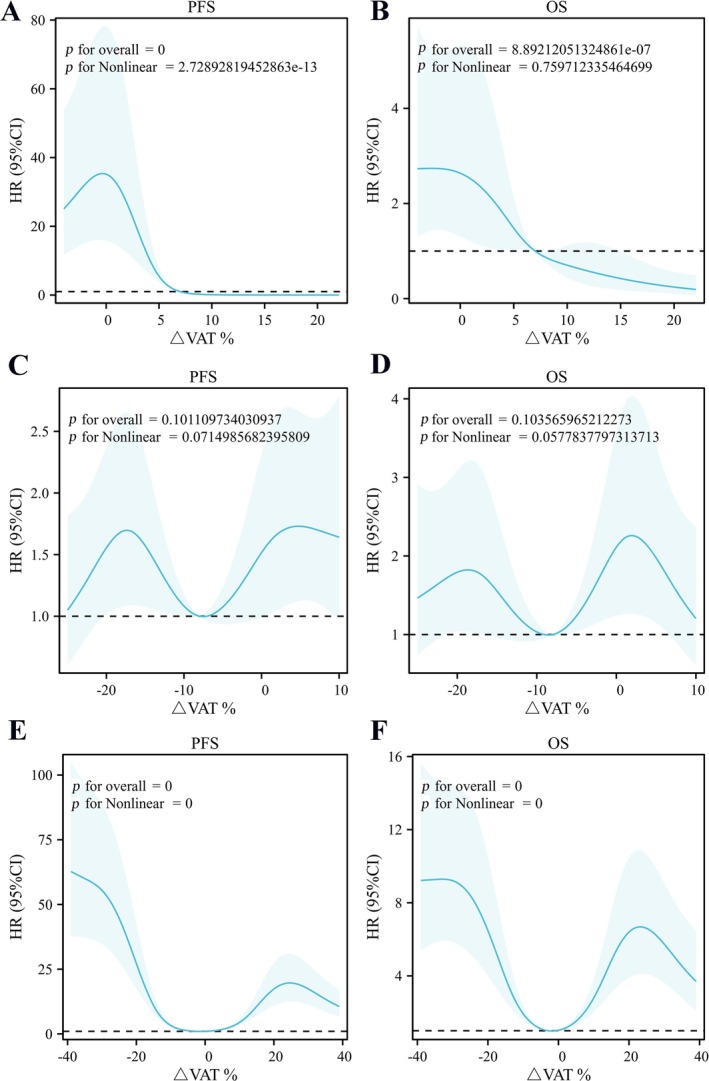
RCS (multivariate model) was used to analyse the relationship between ΔVAT% and PFS/OS. A showed a nonlinear relationship between PFS and ΔVAT% in low VAT group; B showed a linear relationship between OS and ΔVAT% in low VAT group; C showed no relationship between PFS and ΔVAT% in normal VAT group; D showed no relationship between OS and ΔVAT% in normal VAT group; E showed a nonlinear relationship between PFS and ΔVAT% in VO group; F showed a nonlinear relationship between OS and ΔVAT% in VO group. RCS: restricted cubic spline; VAT: visceral adipose tissue.

After NAC treatment of CRLM, the VAT of the overall cohort was 73.00 (31.00–145.00) cm^2^, with an average decrease of 2.64 ± 16.90% compared with pre‐NAC levels (*p* = 0.263). Figure 5A,B show that in the low VAT group, △VAT had a nonlinear relationship with PFS (*p* nonlinear < 0.001), and a linear relationship with OS (*p* nonlinear > 0.05). In the normal VAT group, there was no correlation between △VAT and PFS or OS (*p* overall > 0.05) (Figure 5C,D). In the VO group, △VAT had a nonlinear relationship with both PFS and OS (*p* nonlinear < 0.001) (Figure 5E,F).

## 4 Discussion

This study, based on a multicentre real‐world cohort of 1524 patients with initially resectable CRLM, is the first to discover a U‐shaped relationship between baseline VAT and both PFS/OS of CRLM patients undergoing NAC combined with hepatectomy. We introduce the novel concept of low VAT and demonstrate that patients with low VAT or VO have worse PFS and OS compared with those with normal VAT levels. Furthermore, changes in VAT following NAC differentially impact outcomes depending on the baseline VAT levels. These findings underscore the potential predictive role of VAT in this specific patient population and support including VAT as a component of personalized treatment strategies.

VAT has emerged as an important research focus in cancer [26]. VAT is not merely an organ for fat storage but also plays immunoregulatory and metabolic roles, significantly influencing development, progression and treatment response of cancer [27, 28]. The significant differences in response to NAC among CRLM patients highlight the need for new predictive methods to efficiently and conveniently identify those most likely to benefit from NAC. This study advances previous research by introducing the concept of low VAT. We confirm that the relationship between VAT and CRLM prognosis is not linear, consistent with recent findings [8]. The body roundness index in a study of 32 995 US adults was found to have a U‐shaped association with all‐cause mortality, further supporting our findings [8]. However, our study focuses on a unique population—CRLM patients undergoing NAC combined with hepatectomy. Compared with the body roundness index, VAT is simpler and quicker to assess.

A data analysis of 21 149 CRC patients found that those with low BMI had the highest risk of disease progression and death [15], similar to our findings. For the first time, we identify the predictive role of low VAT in cancer patients undergoing NAC. Interestingly, excessively high BMI did not lead to disease progression [15], possibly explained by the obesity paradox [29]. This further confirms that VAT is a more accurate predictor of VO than BMI. The reasons for the poor prognosis in CRLM patients with low VAT are multifaceted, involving metabolic, immune and nutritional factors. These include the following: (1) VAT provides nutritional support [30]. Low VAT may lead to AEs during chemotherapy and weaken recovery ability, thus affecting prognosis (Figure 3). (2) The rich immune cells in VAT influence immune responses [31, 32, 33]. The low VAT may impair immune function, reducing the ability to fight infections and other complications. Low VAT can potentially be an indicator of malnutrition/decreased appetite. Malnutrition often associated with decreased appetite can lead to a state of chronic inflammation and impaired immune function [34]. Patients with low protein intake may have reduced levels of immunoglobulins, which are crucial for fighting infections. In the context of CRLM, a weakened immune system may be less effective in controlling tumour growth and progression. Malnutrition can disrupt normal metabolic pathways, leading to reduced energy availability for the body to respond to chemotherapy. Moreover, a weakened immune system may create a tumour microenvironment more conducive to cancer cell growth and metastasis. Subsequent analyses also found that low VAT was associated with poorer chemotherapy response and more postoperative complications (Figure 3). (3) Adipocytes and immune cells in VAT secrete various cytokines [32, 35]. Under normal levels, anti‐inflammatory factors help maintain immune system balance. When VAT is too low, this balance may be disrupted, leading to a lack of sufficient anti‐inflammatory response. (4) Low VAT is often accompanied by muscle loss [36]. The imbalance in body composition can weaken the ability to cope with chemotherapy and surgical stress. The potential mechanisms behind the poor prognosis in cancer patients with low VAT are complex, suggesting that attention should be paid not only to the risks of VO but also to the risks associated with excessively low VAT during cancer treatment. Clinicians should take active measures to improve the nutritional and immune status of low VAT patients. Additionally, this suggests that VAT could be included in malnutrition assessments [30]. Although the sample size of our study was sufficient to evaluate the optimal cut‐off value for low VAT, further research is required to determine whether this threshold is applicable to other types of cancer.

The immunological, nutritional and metabolic functions of VAT explain why both extremes of VAT levels lead to poor outcomes in CRLM patients. As a global public health issue, VO increases the risk of developing CRC [37]. VO is associated not only with the increased risk of various metabolic diseases [38, 39] but also with the prognosis of CRC patients by regulating hormone levels, chronic inflammation and driving cytokine production [23, 40, 41, 42]. Unlike CRC, CRLM patients have a higher tumour burden and worse oncological behaviour, making the predictive role of VO in NAC for CRLM particularly significant. Our study reaffirms that VO worsens CRLM prognosis, consistent with previous research findings [13, 14, 23]. This is also the first time this has been confirmed in CRLM patients undergoing NAC. Chronic inflammation, insulin resistance, immune dysregulation and oxidative stress caused by VO may contribute to insensitivity of NAC and tumour progression in CRLM [43, 44]. These highlight the critical role of optimal body composition in cancer prognosis. Adjusting body composition to an optimal ratio before treatment could enhance the ability to cope with chemotherapy and surgical stress. In addition, there are currently automated whole‐body composition calculators available in the CT industry [45], especially with the help of AI. Future RCT studies should consider the use of these automated whole‐body composition calculators.

Unlike other studies that have examined the relationship between body composition and cancer, this study observes the impact of △VAT on prognosis after grouping patients by baseline VAT, aligning with the concept of personalized treatment and precision medicine [46]. Monitoring ΔVAT during treatment through abdominal imaging is convenient. We observed that ΔVAT had different effects on prognosis across different VAT groups, further emphasizing the importance of considering VAT as a dynamic marker. For VO patients, both increases and decreases in ΔVAT worsened prognosis, a complex phenomenon involving multiple physiological and metabolic mechanisms. VAT increase may exacerbate obesity‐related pro‐tumour mechanisms. Studies have found that breast cancer patients who gained ≥ 10% visceral adipose tissue had significantly worse PFS than those who gained < 10% (50). Additionally, for the VO group, loss of VAT may be related to malnutrition and loss of body reserves. This may suggest that body composition may have varying roles at different clinical stages of cancer (50), with specific mechanisms requiring further investigation. Maintaining metabolic stability, optimizing nutritional support and precisely managing body weight and composition during treatment are crucial. In contrast, in patients with low VAT, prognosis gradually improved as ΔVAT increased, reflecting an improvement in their nutritional status, energy reserves and overall health. For the normal VAT, changes in ΔVAT did not alter prognosis, suggesting that individuals in optimal body condition can withstand the dual stress of chemotherapy and surgery. This supports the inclusion of VAT in personalized medicine and highlights the importance of adjusting VAT to an optimal level before treatment. These underscore the potential of VAT as a tool for refining CRLM risk stratification and individualized treatment strategies. Furthermore, changes in ΔVAT during NAC may have prognostic value, indicating that dynamic monitoring of VAT could be valuable in developing treatment plans. Because of the catabolic effects of chemotherapy drugs, prolonged NAC can lead to the loss of VAT. VAT has been shown to play a role in tumour‐microenvironment interactions [12, 47]. A significant reduction in VAT may disrupt these interactions, potentially leading to more aggressive tumour behaviour. Moreover, adipose tissue serves as a crucial energy reserve, and the loss of VAT may negatively impact functional status. Future research should explore how to balance the cycles of NAC, ΔVAT and long‐term prognosis.

Despite providing new insights, this study has several limitations. First, a retrospective study may introduce selection bias, as it relies on pre‐existing imaging data. Second, the study population consisted of patients from five tertiary medical centres in China. Although the sample size was sufficient for the primary analysis, it may not represent a broader CRLM patient population. The differences in clinical practices among centres, such as imaging assessment standards, surgical techniques and postoperative management, may introduce heterogeneity. These variances can potentially affect the relationship between VAT and prognosis. We have standardized the data collection process across centres. And these limitations should still be considered when interpreting the results of our study, and future research may need to further explore and address these issues. Furthermore, although VAT at L3 may be a proxy for overall obesity, future studies should include other body components, such as muscle. Finally, our study did not account for other potential confounding factors that could influence VAT and cancer outcomes, such as KRAS mutations and lifestyle factors.

In conclusion, this multicentre cohort study is the first to reveal a U‐shaped relationship between baseline VAT and prognosis in CRLM patients receiving NAC. Both low VAT and VO are associated with poorer PFS and OS. VAT should be considered an important marker for risk stratification and personalized treatment in CRLM patients, particularly in the dynamic monitoring and management of VAT changes. By identifying patients with optimal VAT levels, clinicians can better tailor individualized treatment strategies, potentially improving outcomes and minimizing unnecessary interventions. Future studies with larger, more diverse populations, standardized imaging protocols and comprehensive data on potential confounders are needed to validate our findings.

## Ethics Statement

Approval of the research protocol by the Institutional Reviewer Board of Fujian Provincial Hospital, The First Affiliated Hospital of Fujian Medical University, Zhejiang Provincial People's Hospital, Hangzhou Red Cross Hospital and The First Hospital of PuTian City. This study has been examined by the ethics committee and have therefore been performed in accordance with the ethical standards laid down in the Declaration of Helsinki.

## Consent

All authors had full access to all the data in the study and had final responsibility for the decision to submit for publication.

## Conflicts of Interest

The authors declare no conflicts of interest.

## Supporting information


**Figure S1** Calculation of the best cut‐off value.

## Data Availability

Data for this study may be requested from the corresponding author where appropriate.
