# Supplementary figures and images for: Low Visceral Adipose Tissue Predicts the Outcome of Neoadjuvant Chemotherapy for Colorectal Liver Metastases: A Multicentre Real‐World Study
Source: J Cachexia Sarcopenia Muscle. 2025 Apr 1;16(2):e13785. doi: 10.1002/jcsm.13785 (PMC11961552; doi:10.1002/jcsm.13785)

Supplemental Figure 1. Calculation of the best cut-off value


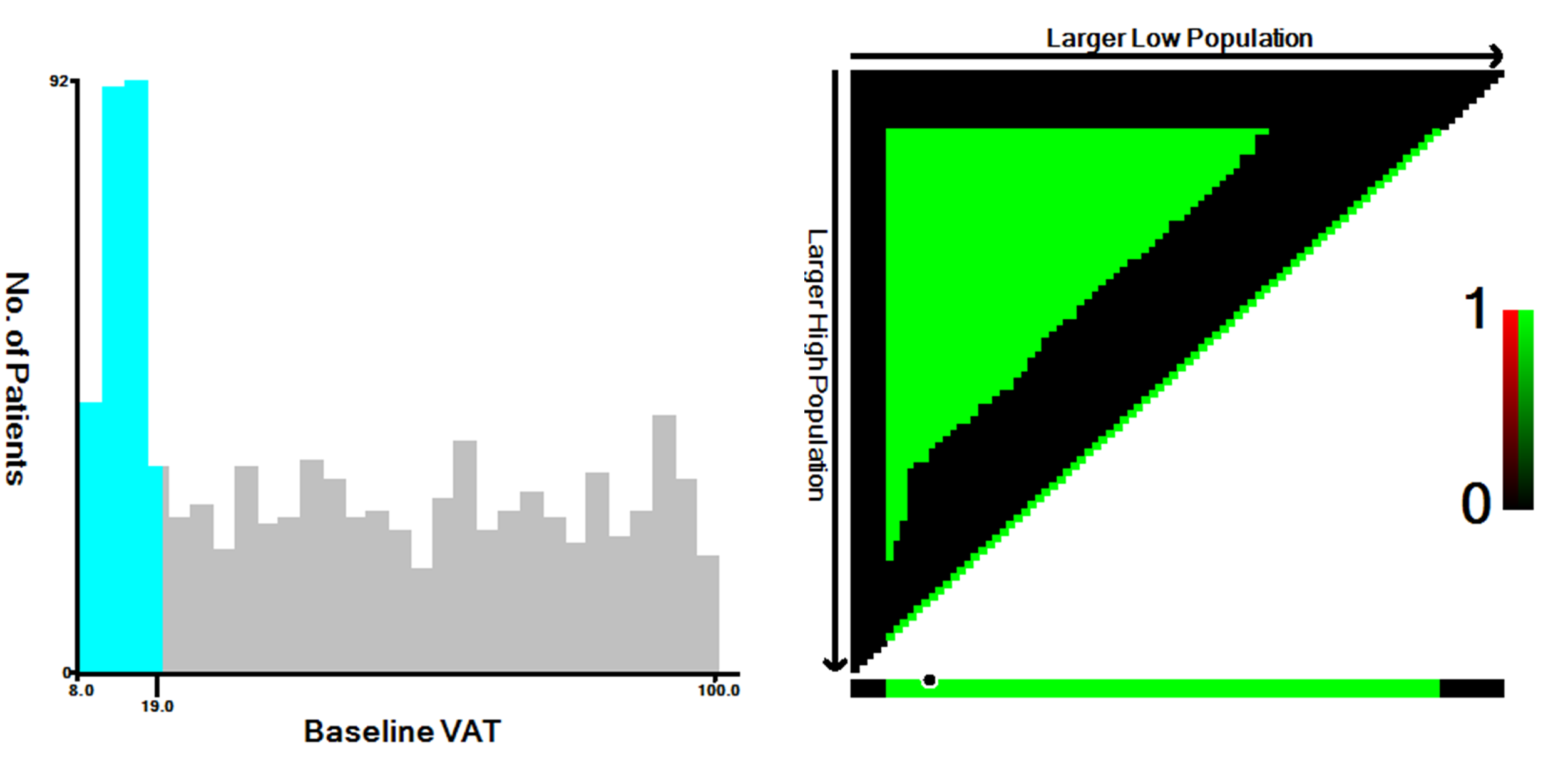


VAT: Visceral adipose tissue.

Supplement: Supplementary file 1 — Figure S1 Calculation of the best cut‐off value. [file JCSM-16-e13785-s001.docx]
